# Updated CDC Recommendations for Using Artemether-Lumefantrine for the Treatment of Uncomplicated Malaria in Pregnant Women in the United States

**DOI:** 10.15585/mmwr.mm6714a4

**Published:** 2018-04-13

**Authors:** Sarah-Blythe Ballard, Allison Salinger, MPHc, Paul M. Arguin, Meghna Desai, Kathrine R. Tan

**Affiliations:** ^1^Epidemic Intelligence Service, CDC; ^2^Division of Parasitic Diseases and Malaria, Center for Global Health, CDC; ^3^Hubert Department of Global Health, Emory University, Atlanta, Georgia.

Malaria infection during pregnancy is associated with an increased risk for maternal and fetal complications. In the United States, treatment options for uncomplicated, chloroquine-resistant *Plasmodium falciparum* and *P. vivax* malaria in pregnant women are limited to mefloquine or quinine plus clindamycin (*1*). However, limited availability of quinine and increasing resistance to mefloquine restrict these options. Strong evidence now demonstrates that artemether-lumefantrine (AL) (Coartem) is effective and safe in the treatment of malaria in pregnancy. The World Health Organization (WHO) has endorsed artemisinin-based combination therapies (ACTs), such as AL, for treatment of uncomplicated malaria during the second and third trimesters of pregnancy and is currently considering whether to add ACTs, including AL, as an option for malaria treatment during the first trimester (*2*,*3*). This policy note reviews the evidence and updates CDC recommendations to include AL as a treatment option for uncomplicated malaria during the second and third trimesters of pregnancy and during the first trimester of pregnancy when other treatment options are unavailable. These updated recommendations reflect current evidence and are consistent with WHO treatment guidelines.

## Background

Each year, approximately 1,700 cases of imported malaria occur in the United States; approximately 630 (37%) of these cases occur in women, including 5%–6% who are pregnant at the time they are infected (*4*). Treatment options for uncomplicated, chloroquine-resistant *P. falciparum* and *P. vivax* malaria infections in pregnant women in the United States are threatened by the spread of mefloquine resistance in Southeast Asia. Having only one quinine and mefloquine manufacturer in the United States can adversely affect access. In 2009, the Food and Drug Administration (FDA) approved AL for the treatment of uncomplicated malaria. At that time, this combination was not approved for use in pregnancy because animal research data indicated a potential association with poor pregnancy outcomes, and insufficient human data were available. Since then, global experience has contributed substantial evidence of the safety and efficacy of AL throughout pregnancy. Given the need for an additional option to treat uncomplicated malaria in pregnant women in the United States, a systematic review of the literature was performed to evaluate the safety and efficacy of AL use during pregnancy, and findings were used to update CDC recommendations.

## Methods

A systematic review of English-language research articles listed in PubMed was conducted using the keywords “artemether,” “lumefantrine,” “Coartem,” and “malaria in pregnancy.” Clinical trials, observational studies, meta-analyses, and case reports of uncomplicated malaria treatment during pregnancy were included. Studies that did not include treatment or pregnancy outcomes were excluded, as were studies that did not identify the trimester of treatment. Review article and meta-analysis references were examined for additional primary source articles for inclusion. Online search results were compiled and deduplicated. Two independent reviewers determined the relevance of each article to the research objective based first on title, then abstract, then full text. If reviewers had discordant findings from title or abstract review, the article was included in the next review phase. The following data were abstracted and reviewed: participant age; geographic location; parity; reason for drug treatment (uncomplicated versus severe malaria); trimesters during which treatment occurred; medication dose administered; treatment duration; treatment outcomes; and pregnancy outcomes, which included miscarriage (pregnancy loss at <28 weeks’ gestation), stillbirth (pregnancy loss at ≥28 weeks’ gestation), preterm birth (<37 weeks’ gestation), low birth weight (<2,500 g), congenital abnormalities, and any maternal adverse events reported.

## Rationale and Evidence

**Systematic review results.** In the initial search, 1,726 articles were identified. After excluding four articles during deduplication, 1,534 during title review, 94 during abstract review, and 73 after full text review, 21 articles remained and were included in the review.

**Efficacy.** One meta-analysis (*5*) and five randomized open-label controlled trials performed in Uganda and Thailand examined the efficacy of ACTs for uncomplicated *P. falciparum* in women during their second and third trimesters of pregnancy and found cure rates ≥94.9%, with ACTs performing equal to or better than quinine-based regimens (Table 1) (*6*–*10*). A meta-analysis of African and Asian studies found lower but statistically similar treatment failure rates by days 28–63 in women taking ACTs versus non-ACTs to treat uncomplicated malaria in the second and third trimesters of pregnancy (pooled risk ratio random effects = 0.41; 95% confidence interval (CI) = 0.16–1.06; six trials) (*5*). With respect to AL efficacy during the second and third trimesters of pregnancy, a concern existed that a reduction in relative bioavailability of lumefantrine in pregnant women might affect treatment success later in pregnancy (*11*–*15*). However, the evidence presented indicates that treatment in pregnancy is efficacious at the doses currently recommended for nonpregnant women.

**TABLE 1:** Findings of randomized trials of artemisinin-based regimens for treatment of malaria in pregnancy

Author, publication year	Country	Indication for treatment	Drug regimen	No. of participants	Follow-up time (days)	Treatment outcome, % (95% CI)
McGready, et al., 2000*	Thailand	Uncomplicated *P. falciparum*, second and third trimesters	1. MQ 25 mg/kg x 1 and As 4 mg/kg/d x 3d	66	63	Cure 98.2 (94.7–100)^†^
			2. Q 10 mg/kg q8hr x 7d	42	63	Cure 67.0 (43.3–90.8)^†^
McGready, et al., 2001^§^	Thailand	Uncomplicated *P. falciparum*, second and third trimesters	1. As 2 mg/kg/d x 7d	64	42	Cure 100
			2. Q 10 mg/kg q8hr x7d and CL 5 mg/kg q8hr x7d	65	42	Cure 100
McGready, et al., 2005^¶^	Thailand	Uncomplicated *P. falciparum*, second and third trimesters	As 4 mg/kg/d x 3d and A 20 mg/kg/d x 3d and P 8 mg/kg/d x 3d	39	63	Cure 94.9 (81.37–99.11)^†,^**
			Q 10 mg/kg q8hr x 7d	42	63	Cure 63.4 (46.9–77.4)^†,††^
Piola, et al., 2010^§§^	Uganda	Uncomplicated *P. falciparum*	1. AL 20/120 mg 4 tabs at 0 and 8hr x 1d, then BID x 2d	152	42	Cure 99.3 (96.0–99.9)^†,¶¶^
			2. Q 10 mg/kg q8hr x 7d	152	42	Cure 97.6 (93.1–99.5)^†,^***
Kaye, et al., 2008^†††^	Uganda	Uncomplicated *P. falciparum*, second and third trimesters	1. AL 20/120 mg 4 tabs at 0 and 8hr x1d, then BID x 2d	57	28	Cure 100
			2. LapDap x 3d	57	28	Cure 100

**Abbreviations:** A = atovaquone; AL = artemether-lumefantrine; AQ = amodiaquine; As = artesunate; BID = twice daily; CI = confidence interval; d = days; hr = hour(s); kg = kilogram; LapDap = chlorproguanil-dapsone; mg = milligram; MQ = mefloquine; P = proguanil; PCR = polymerase chain reaction; Q = quinine; qd = once daily; q8hr = every 8 hours.

* McGready R, Brockman A, Cho T, et al. Randomized comparison of mefloquine-artesunate versus quinine in the treatment of multidrug-resistant falciparum malaria in pregnancy. Trans R Soc Trop Med Hyg 2000;94:689–93.

^†^ PCR-adjusted.

^§^ McGready R, Cho T, Keo NK, et al. Artemisinin antimalarials in pregnancy: a prospective treatment study of 539 episodes of multidrug-resistant *Plasmodium falciparum*. Clin Infect Dis 2001;33:2009–16.

^¶^ McGready R, Ashley EA, Moo E, et al. A randomized comparison of artesunate-atovaquone-proguanil versus quinine in treatment for uncomplicated falciparum malaria during pregnancy. J Infect Dis 2005;192:846–53.

** 37 of 39 participants.

^††^ 26 of 41 participants.

^§§^ Piola P, Nabasumba C, Turyakira E, et al. Efficacy and safety of artemether-lumefantrine compared with quinine in pregnant women with uncomplicated *Plasmodium falciparum* malaria: an open-label, randomised, non-inferiority trial. Lancet Infect Dis 2010;10:762–9.

^¶¶^ 137 of 139 participants.

*** 122 of 125 participants.

^†††^ Kaye DK, Nshemerirwe R, Mutyaba TS, Ndeezi G. A randomized clinical trial comparing safety, clinical and parasitological response to artemether-lumefantrine and chlorproguanil-dapsone in treatment of uncomplicated malaria in pregnancy in Mulago hospital, Uganda. J Infect Dev Ctries 2008;2:135–9.

**Second and third trimester safety.** Data evaluating pregnancy outcomes in women taking ACTs during the second or third trimesters of pregnancy were available from 16 studies (Table 2). No differences in pregnancy outcomes were identified in four trials comparing ACTs with quinine-based regimens in Uganda and Thailand (*6*,*7*,*9*,*10*), one of which used AL (*9*), and in four other trials comparing AL with other ACTs in Nigeria (two studies), Thailand, and multiple sites in Africa (*16*–*19*). A Zambian cohort study comparing treatment of uncomplicated malaria using AL with treatment using sulfadoxine-pyrimethamine found similar pregnancy outcomes between groups (*20*). In addition, two meta-analyses of women with malaria in the second and third trimester of pregnancy found no association between ACT treatment and congenital malformations or miscarriage (*5*,*21*). Overall, fewer maternal adverse events occurred among women taking ACTs than among those taking non-ACTs (Table 2). One trial in Thailand found a relatively higher proportion of day 7 anemia among those treated with mefloquine-artesunate (67%) than among those treated with a quinine-based regimen (42%) (*6*). Four trials and one meta-analysis comparing ACTs with quinine-based regimens found that pregnant women taking quinine had higher rates of tinnitus, dizziness, and vomiting than did pregnant women taking ACTs (*5*–*9*). The three trials comparing AL with other ACTs found no differences in rates of serious adverse maternal effects between groups (*9*,*16*,*18*).

**TABLE 2:** Summary of studies using artemisinin-based treatment for malaria in second and third trimesters of pregnancy and safety outcomes

Author, publication year	Indication (country)	Drug(s)	No. of participants	Pregnancy outcomes, n/N (%)*	Congenital anomalies, n/N (%)	Maternal adverse events, n/N (%)
Randomized trials (all open label) using nonartemisinin drug in comparison group						
McGready, et al., 2000^†^	Uncomplicated *P. falciparum* (Thailand)	MQ 25 mg/kg x 1 and As 4 mg/kg/d x 3d	66	Miscarriage, 2 (3)	0 (0)	Anemia day 7, 32/48 (67)^§^
				Stillbirth, 0 (0)		Dizziness, (45)^§^
				Low birth weight, 9/53 (17)		Tinnitus, (17)^§^
		Q 10 mg/kg q8hr x 7d	42	Miscarriage, 0 (0)	0 (0)	Anemia day 7, 14/33 (42)^§^
				Stillbirths, 0 (0)		Dizziness, (87)^§^
				Low birth weight, 6/33 (18)		Tinnitus, (66)^§^
McGready, et al., 2001^¶^	Uncomplicated *P. falciparum* (Thailand)	As 2 mg/kg/d x 7d	64	Stillbirth, 1 (2)**	Minor, 1 (2)	Tinnitus, (9)^§^
		Q 10 mg/kg q8hr x 7d and CL 5 mg/kg q8hr x 7d	65	Stillbirth, 1 (2)**	Major, 1(2)	Tinnitus, (45)^§^
McGready, et al., 2005^††,§§^	Uncomplicated *P. falciparum* (Thailand)	As 4 mg/kg/d x 3d and A 20 mg/ kg/d x 3d and P 8 mg/kg/d x 3d	39	Preterm, 4/34 (12)	Polythelia and cleft lip and palate, 2/34 (6)**	Tinnitus, (24)^§^
				Low birth weight, 6/23 (26)		
		Q 10 mg/kg q8hr x 7d	42	Stillbirth, 1 (2)	Left aural atresia, 1/38 (3)**	Tinnitus, (79)^§^
				Preterm, 6/38 (16)		
				Low birth weight, 4/30 (13)		
Piola, et al., 2010^¶¶^	Uncomplicated *P. falciparum* (Uganda)	AL 20/120 mg 4 tabs at 0 and 8hr x 1d, then BID x 2d	152	Miscarriage, 2/144 (1)	Polydactyly, 2 (1)**	Tinnitus, 0 (0)^§^
				Intrauterine fetal death, 1/144 (1)	Acyanotic heart disease, 1 (1)	Headache, 26 (17)^§^
				Stillbirth, 2/144 (1)		Nausea, 8 (5)^§^
				Preterm, 12/143 (1)		Vomiting, 6 (4)^§^
				Low birth weight, 12/120 (10)		Anorexia, 6 (4)^§^
		Q 10 mg/kg q8hr x 7d	152	Miscarriage, 2/137 (2)	Polydactyly, 2 (1)**	Tinnitus, 111 (73)^§^
				Intrauterine fetal death, 2/137 (2)		Headache, 9 (6)^§^
				Stillbirth, 3/137 (2)		Nausea, 26 (17)^§^
				Preterm, 17/137 (3)		Vomiting, 28 (18)^§^
				Low birth weight, 16/137 (13)		Anorexia, 16 (11)^§^
Kaye, et al., 2008***	Uncomplicated *P. falciparum* (Uganda)	AL 20/120 mg 4 tabs at 0 and 8hr x 1d, then BID x 2d	57	Not assessed	Not assessed	Palpitations, 4 (7)
						Dizziness, 1 (2)
						Drowsiness, 1 (2)
						Rash, 1 (2)
		LapDap x 3d	57	Not assessed	Not assessed	Vomiting, 1 (2)
						Diarrhea, 1 (2)
						Palpitations, 1 (2)
**Randomized trials (open label unless otherwise noted) using artemisinin in comparison group**						
Sowunmi, et al., 1998^†††^	Failed CQ, SP or CQ-SP treatment for *P. falciparum* (Nigeria)	Ar 3.2 mg/kg IM x 1 then 1.6 mg/kg IM qd x 4d	23	IUGR, 1	None	None
		Ar 3.2 mg/kg IM x 1 then MQ 7.5 mg/kg qd x 2d	22	None	None	Abdominal discomfort, 2 (9)
						Dizziness, 2 (9)
McGready, et al., 2008^§§§^	Uncomplicated *P. falciparum* (Thailand)	AL 20/120 mg 4 tabs BID x 3d	124	Miscarriage, 0 (0)	None	Vomiting, 2 (2)
				Stillbirth, 1/119 (1)		
		As 2 mg/kg qd x 7d	125	Miscarriage, 1/122 (1)**	None	Vomiting, 1 (1)
				Stillbirth, 1/119 (1)		Rash, 1 (1)
Ukah, et al., 2015^¶¶¶^	Uncomplicated *P. falciparum* (Nigeria, double-blind)	AL (80 mg/480 mg) BID x 3d	75	Miscarriage, 1/71 (1)	Not assessed	Body weakness 2 (3)
				Stillbirth, 2/71 (3)		Pruritis 0 (0)
		Ar-AQ (100 mg/270 mg) BID x 3d	75	Miscarriage, 1/65 (2)	Not assessed	Body weakness, 26 (35)
				Stillbirth, 1/65 (2)		Pruritis, 4 (5)
PREGACT, 2016****	*P. falciparum* (four African countries)	AL	880	Miscarriage, 1	Any defect, 17/832 (2)	
				Stillbirth, 16/856 (2)		
				Preterm, (10)		
		AQ-As	842	Miscarriage, 4 (<1)	Any defect, 8/776 (1)	Anemia, 2 (<1
				Stillbirth, 17/815 (2)		Abdominal pain, 1 (<1)
				Preterm, (3)		Malaise, 2 (<1)
		MQ-As	848	Miscarriage, 4	Any defect, 13/780 (2)	Abdominal pain, 1 (<1)
				Stillbirth, 23/821 (3)		Vomiting, 2 (<1)
				Preterm, (8)		Malaise, 1 (<1)
		DHA-PIP	853	Miscarriage, 4 (<1)	Any defect, 6/767 (1)	Headache/weakness, 1 (<1)
				Stillbirth, 22/818 (3)		
				Preterm, (10)		
Cohort study						
Manyando, et al., 2010^††††,§§§§^	Uncomplicated *P. falciparum* (Zambia)	AL 20 mg/120 mg 4 tabs BID x 3d	495	Miscarriage, 7/504 (1) (all first trimester exposures)	Any defect, 29/449 (7)	Not reported
				Stillbirth, 9/504 (2)		
				Preterm, 71/504 (14)		
		SP (1500 mg/75 mg)	506	Miscarriage, 8/516 (2) (in 5 women, including one with twins and one with triplets)	Any defect, 18/444 (4)	Not reported
				Stillbirth, 13/516 (3)		
				Preterm, 90/516 (17)		
**Descriptive studies (includes pharmacokinetic studies and case series)**						
McGready, et al., 2001^¶^ (includes data published 1998)^¶¶¶¶^	*P. falciparum* or mixed, primary and recrudescent, uncomplicated and severe (Thailand)	As given 2–4 mg/kg up to 7 days (varies by indication) or As 4 mg/kg qd x 3d and AP or As 4 mg/kg qd x 3d and MQ	461	Miscarriage, 20/414 (5)	Any defect, 3/386 (1) Major 1/386 (0)	No serious adverse events
				Stillbirth, 7/386 (2)		
		Community (no treatment)		Miscarriage, 1003/8154 (12)	Any defect, 866/6418 (14)	
				Stillbirth, 114/7058 (2)		
				Low birth weight, 866/6418 (14)		
Mosha, et al., 2014*****	Uncomplicated *P. falciparum* (Tanzania)	AL 20/120 mg 4 tabs at 0 and 8hr x1d, then BID x 2d	35	Not assessed, (follow-up to 42 days only)	Not assessed	No serious adverse events
Nyunt, et al., 2015^†††††^	Uncomplicated *P. falciparum* (Uganda)	AL 20/120 mg 4 tabs at 0 and 8hr x 1d, then BID x 2d	30	Not assessed, (follow-up to 42 days only)	Not assessed	No serious adverse events
Adam, et al., 2004^§§§§§,¶¶¶¶¶^	*P. falciparum* (Sudan)	Ar 80 mg IM BID x1d then qd x 2d	28	Miscarriage, 0 (0)	Not assessed	Not assessed
				Stillbirth, 0 (0)		
				Premature, 1 (4)**		
Adam, et al., 2009******^,¶¶¶¶¶^	*P. falciparum* (Sudan)	Ar IM	62	Miscarriage, 2 (3)** (both had received artemether injections early in pregnancy and miscarried while receiving quinine infusions for a second malaria infection)	Not assessed	Not assessed
		As and SP				
		AL				
Wang, 1981^††††††^	*“Plasmodium”* (China)	Ar in oil 500–900 mg IM qd x 3d or Ar 600 mg IM qd x 3d	6	Miscarriage, 0 (0)	Any defect, 0 (0)	Not assessed
				Stillbirth, 0 (0)		
				Premature, 0 (0)		

**Abbreviations:** A = atovaquone; AL = artemether-lumefantrine; AQ = amodiaquine; Ar = artemether; As = artesunate; BID = twice daily; CL = clindamycin; CQ = chloroquine; d = day(s); DHA-PIP = dihydroartemisinin-piperaquine; hr = hour(s); IM = intramuscular; IUGR = intrauterine growth retardation; kg = kilogram; LapDap = chlorproguanil-dapsone; mg = milligram; MQ = mefloquine; P = proguanil; Q = quinine; qd = once daily; q8hr = every 8 hours; SP = sulfadoxine pyrimethamine.

* In studies with incomplete outcome data, denominators are provided.

^†^ McGready R, Brockman A, Cho T, et al. Randomized comparison of mefloquine-artesunate versus quinine in the treatment of multidrug-resistant falciparum malaria in pregnancy. Trans R Soc Trop Med Hyg 2000;94:689–93.

^§^ Significant difference between comparison groups.

^¶^ McGready R, Cho T, Keo NK, et al. Artemisinin antimalarials in pregnancy: a prospective treatment study of 539 episodes of multidrug-resistant *Plasmodium falciparum*. Clin Infect Dis 2001;33:2009–16.

** Considered not related to drug.

^††^ McGready R, Ashley EA, Moo E, et al. A randomized comparison of artesunate-atovaquone-proguanil versus quinine in treatment for uncomplicated falciparum malaria during pregnancy. J Infect Dis 2005;192:846–53.

^§§^ 1-year follow-up of infants indicated no differences in development.

^¶¶^ Piola P, Nabasumba C, Turyakira E, et al. Efficacy and safety of artemether-lumefantrine compared with quinine in pregnant women with uncomplicated *Plasmodium falciparum* malaria: an open-label, randomised, non-inferiority trial. Lancet Infect Dis 2010;10:762–9.

*** Kaye DK, Nshemerirwe R, Mutyaba TS, Ndeezi G. A randomized clinical trial comparing safety, clinical and parasitological response to artemether-lumefantrine and chlorproguanil-dapsone in treatment of uncomplicated malaria in pregnancy in Mulago hospital, Uganda. J Infect Dev Ctries 2008;2:135–9.

^†††^ Sowunmi A, Oduola AMJ, Ogundahunsi OAT, et al. Randomised trial of artemether versus artemether and mefloquine for the treatment of chloroquine/sufadoxine-pyrimethamine-resistant falciparum malaria during pregnancy. J Obstet Gynaecol 1998;18:322–7.

^§§§^ McGready R, Tan SO, Ashley EA, et al. A randomised controlled trial of artemether-lumefantrine versus artesunate for uncomplicated *Plasmodium falciparum* treatment in pregnancy. Rogerson S, editor. PLoS Med 2008; 5:e253.

^¶¶¶^ Ukah M, Badejoko O, Ogunniyi S, Loto O, Aboderin O, Fatusi A. A randomized trial of artesunate-amodiaquine versus artemether-lumefantrine for the treatment of acute uncomplicated malaria in pregnancy. Int J Gynaecol Obstet 2015;131:41–4.

**** PREGACT Study Group. Four artemisinin-based treatments in African pregnant women with malaria. N Engl J Med 2016;374:913–27.

^††††^ Manyando C, Njunju EM, Virtanen M, Hamed K, Gomes M, Van Geertruyden JP. Exposure to artemether-lumefantrine (Coartem) in first trimester pregnancy in an observational study in Zambia. Malar J 2015;14:77.

^§§§§^ Included women at all trimesters.

^¶¶¶¶^ McGready R, Cho T, Cho JJ, et al. Artemisinin derivatives in the treatment of falciparum malaria in pregnancy. Trans R Soc Trop Med Hyg 1998;92:430–3.

***** Mosha D, Mazuguni F, Mrema S, Sevene E, Abdulla S, Genton B. Safety of artemether-lumefantrine exposure in first trimester of pregnancy: an observational cohort. Malar J 2014;13:197.

^†††††^ Nyunt MM, Nguyen VK, Kajubi R, et al. Artemether-lumefantrine pharmacokinetics and clinical response are minimally altered in pregnant Ugandan women treated for uncomplicated falciparum malaria. Antimicrob Agents Chemother 2016;60:1274–82.

^§§§§§^ Adam I, Elwasila E, Mohammed Ali DA, Elansari E, Elbashir MI. Artemether in the treatment of falciparum malaria during pregnancy in eastern Sudan. Trans R Soc Trop Med Hyg 2004;98:509–13.

^¶¶¶¶¶^ Included women in first and second trimesters.

****** Adam I, Elhassan EM, Omer EM, Abdulla MA, Mahgoub HM, Adam GK. Safety of artemisinins during early pregnancy, assessed in 62 Sudanese women. Ann Trop Med Parasitol 2009;103:205–10.

^††††††^ Wang TY. Follow-up observation on the therapeutic effects and remote reactions of artemisinin (Qinghaosu) and artemether in treating malaria in pregnant woman. J Tradit Chin Med 1989;9:28–30.

**First trimester safety.** No randomized trials evaluating AL use during the first trimester of pregnancy were found (Table 3). However, a meta-analysis of observational and other studies from six sub-Saharan African countries and the Thai-Burmese border included data from a total of 717 women taking ACTs during the first trimester of pregnancy (*22*). Comparisons of pregnancy outcomes between women taking ACTs and those receiving a quinine-based regimen anytime during the first trimester and treatment with ACTs versus quinine-based regimen during 6–12 weeks’ gestational age demonstrated no differences in miscarriage, stillbirth, or pregnancy loss (miscarriage and still birth combined) for women treated with ACTs versus quinine-based regimens during either period. Although limited by sample size, the pooled prevalences of congenital anomalies in infants born to mothers taking ACTs versus quinine-based regimens in the first trimester were similar (1.5%, 95% CI = 0.6–3.5 versus 1.2%, 95% CI = 0.6–2.4, respectively) (*22*).

**TABLE 3:** Summary of safety outcomes in studies using artemisinin-based treatment for malaria in first trimester of pregnancy

Author, publication year	Description or indication (country)	Drug or regimen (no.)	Pregnancy outcomes, no. (%) (unless otherwise indicated)*	Congenital anomalies, no. (%) (unless otherwise indicated)	Maternal adverse events, no. (%) (unless otherwise indicated)
**Meta-analysis**					
Dellicour, et al., 2017^†^	Included five observational studies (individual participant data from six sub-Saharan African countries, and aggregate data from Thailand)	Areg (717)	Miscarriage:	As 1.5% (95% CI = 0.6–3.5); Q 1.2% (95% CI = 0.6–2.4)	Not assessed
			Areg versus Q: aHR = 0.73 (95% CI = 0.44–1.21)		
			Areg versus none: aHR = 1.16 (95% CI = 0.81–1.66)		
		Q (947)	Stillbirth:		
			Areg versus Q: aHR = 0.29 (95% CI = 0.08–1.02)		
			Areg versus none: aHR = 0.65 (95% CI = 0.34–1.23)		
		No antimalarials (28,954)	Stillbirth and miscarriage:		
			Areg versus Q: aHR = 0.58 (95% CI = 0.36–1.02)		

Observational studies					
Any anomaly, 1 (1) Any anomaly, 1 (1) Any anomaly, 2 (3)	Identified women with inadvertent use of AL, other antimalarials, or none, then followed to birth outcome (Tanzania)	AL (164)	Miscarriage, 5 (3) and stillbirth, 6 (3.7);		Not assessed
			aOR = 1.4 (95% CI = 0.8–2.5, p = 0.295)		
			Low birth weight, 8 (5.2);		
			aOR = 1.2 (95% CI = 0.6–2.5, p = 0.573)		
			Preterm, 8 (5.2);		
			aOR = 0.9 (95% CI = 0.5–1.8, p = 0.865)		
		Q (70)	Miscarriage, 3(4.3) and stillbirth, 5 (7.1);		Not assessed
			aOR = 2.5 (95% CI = 1.3–5.1, p = 0.009)		
			Low birth weight, 1 (1.6);		
			aOR = 0.6 (0.1–2.4, p = 0.461)		
			Preterm, aOR = 2.6 (95% CI = 1.3– 5.3, p = 0.007)		
		SP (66)	Miscarriage, 0 and stillbirth, 2 (3.0);		Not assessed
			aOR = 0.5 (95% CI = 0.1–2.0, p = 0.312)		
			Low birth weight, 2 (3.1);		
			aOR = 0.7 (95% CI = 0.2–3.0, p = 0.639)		
			Preterm, 7 (10.9);		
			aOR = 1.8 (95% CI = 0.8–4.1, p = 0.160)		
		AQ (11)	Miscarriage, 0 and stillbirth, 0	Any anomaly, 0 (0)	Not assessed
			Low birth weight, 0		
			Preterm, 0		
		No antimalarials (1,464)	Miscarriage, 34 (2.3) and stillbirth, 49 (3.3);	Any anomaly, 19 (1)	Not assessed
			aOR = 0.8 (95% CI = 0.5–1.2, p = 0.260)		
			Low birth weight, 69 (5.0);		
			aOR = 1.2 (95% CI = 0.6–2.3, p = 0.564)		
			Preterm, 88 (6.4);		
			aOR = 0.7 (95% CI = 0.5–1.1, p = 0.168)		
Dellicour, et al., 2015**^,¶^	Identified women with inadvertent use of AL, other antimalarials, or none, then followed to birth outcome (Kenya)	Confirmed ACT (77)	Miscarriage:	Not assessed	Not assessed
		Unconfirmed ACT (222) Q (13)	Confirmed ACT exposure only:		
			ACT 6/77 versus no antimalarial 57/793		
			aHR = 1.72 (95% CI = 0.66–4.45, p = 0.266)		
		No ACT exposure (835)	Q 0/3 versus no antimalarial 57/793		
			ACT 5/72 versus Q 1/13;		
			aHR = 0.48 (95% CI = 0.12–1.89, p = 0.297)		
			Confirmed and unconfirmed ACT: ACT 29/299 versus no antimalarial 57/793;		
			aHR = 1.66 (95% CI = 1.04–2.67, p = 0.034)		
			Q 1/13 versus no antimalarial 57/793;		
			aHR = 4.27 (95% CI = 0.53–34.33, p = 0.172)		
			ACT 28/286 versus Q 1/13;		
			aHR = 0.64 (95% CI = 0.08–4.91, p = 0.665)		
Moore, et al., 2016^††,¶^	Data from antenatal clinics analyzed (Thai-Myanmar border)	Areg (183)	Miscarriage: when compared with Q or Q and CL, Areg, 92 (11): aHR = 0.78 (95% CI = 0.45–1.34, p = 0.3645)	Any malformation: Uncomplicated Pf treated with Areg, 2/109 (2), Q, 9/641 (1), Severe Pf treated with: Areg, 2/22 (9); Q, 0/8 (0)	Not assessed
		MQ (25)	MQ 2 (8): aHR = 0.54 (95% CI = 0.13–2.31, p = 0.4082)		
		Q or Q and CL (971)	When comparing malaria with no malaria in first trimester, miscarriage: aHR = 1.61 (95% CI = 1.32–1.97, p<0.0001)		
Manyando, et al., 2015^§§,¶^	Data analyzed from previous prospective cohort, women with inadvertent first trimester exposure (Zambia)	AL (135)	Miscarriage not assessed	Any malformation, 9 (7)	Not assessed
			Stillbirth, 2 (1.5) (95% CI = 0.4–5.2)		
			Low birth weight, 13 (10.2)		
		AL and SP (7)	Miscarriage not assessed	Any malformation, 8/121 (7)	Not assessed
			Stillbirth, 0 (0) (95% CI = 0–39.0)		
			Low birth weight, 1 (14.3)		
		SP and/or Q (129)	Miscarriage not assessed		Not assessed
			Stillbirth, 3 (2.3) (95% CI = 0.8–6.6)		
			Low birth weight, 8 (6.7)		
		No antimalarial (644)	Miscarriage not assessed	Not assessed	Not assessed
			Stillbirth, 17 (2.6) (95% CI = 1.7–4.2)		
			Low birth weight, 52 (8.7)		
**Descriptive studies**					
McGready, et al., 2001^¶¶^ (includes data from McGready et al., 1998)***	P for mixed infection, both primary and recrudescent, uncomplicated and severe (Thailand)	Areg (19 primary treatment, 25 for retreatment)	Miscarriage, 7 (18.9)^†††^	Any malformation, 0	Not assessed
		Community (no treatment)	Miscarriage, 1,003/8,154 (12.3)	Any malformation, 56/3,707 (2)	Not assessed

**Abbreviations:** A = atovaquone; aHR = adjusted hazard ratio; AL = artemether-lumefantrine; aOR = adjusted odds ratio; AQ = amodiaquine; Ar = artemether; Areg = artemisinin regimen; As = artesunate; BID = twice daily; CI = confidence interval; CL = clindamycin; CQ = chloroquine; DHA-PIP = dihydroartemisinin-piperaquine; LapDap = chlorproguanil-dapsone; MQ = mefloquine; P = proguanil; Pf = *Plasmodium falciparum*; Q = quinine; SP = sulfadoxine pyrimethamine.

* In studies with incomplete outcome data, denominators are provided.

^†^ Dellicour S, Sevene E, McGready R, et al. First-trimester artemisinin derivatives and quinine treatments and the risk of adverse pregnancy outcomes in Africa and Asia: a meta-analysis of observational studies. Krishna S, editor. PLOS Med 2017;14:e1002290.

^§^ Mosha D, Mazuguni F, Mrema S, Sevene E, Abdulla S, Genton B. Safety of artemether-lumefantrine exposure in first trimester of pregnancy: an observational cohort. Malar J 2014;13:197.

^¶^ Study included in Dellicour 2017 meta-analysis.

** Dellicour S, Sevene E, McGready R, et al. First-trimester artemisinin derivatives and quinine treatments and the risk of adverse pregnancy outcomes in Africa and Asia: a meta-analysis of observational studies. Krishna S, editor. PLOS Med 2017;14:e1002290.

^††^ Moore KA, Simpson JA, Paw MK, et al. Safety of artemisinins in first trimester of prospectively followed pregnancies: an observational study. Lancet Infect Dis 2016;16:576–83.

^§§^ Manyando C, Njunju EM, Virtanen M, Hamed K, Gomes M, Van Geertruyden JP. Exposure to artemether-lumefantrine (Coartem) in first trimester pregnancy in an observational study in Zambia. Malar J 2015;14:77.

^¶¶^ McGready R, Cho T, Keo NK, et al. Artemisinin antimalarials in pregnancy: a prospective treatment study of 539 episodes of multidrug-resistant Plasmodium falciparum.

*** McGready R, Cho T, Cho JJ, et al. Artemisinin derivatives in the treatment of falciparum malaria in pregnancy. Trans R Soc Trop Med Hyg 1998;92:430–3.

^†††^ Not different from overall community rate p = 0.211.

## Recommendation

Malaria infection during pregnancy can result in serious maternal and fetal complications. On the basis of the strength and quality of this evidence, CDC recommends AL as an additional option for treatment of uncomplicated malaria in pregnant women in the United States during the second and third trimesters of pregnancy at the same doses recommended for nonpregnant women. Women in the United States with uncomplicated malaria during the first trimester of pregnancy should be treated with the currently recommended options of either mefloquine or quinine plus clindamycin. However, when neither of these options is available, AL should be considered for treatment.

## Discussion

This update of CDC recommendations based on accumulated evidence of the safety of AL in pregnancy is in line with the malaria treatment guidelines of other countries without endemic malaria and WHO (*3*,*23*,*24*). On the basis of the current strength and quality of the first trimester safety and efficacy evidence, the addition of ACTs, including AL, as a first-line treatment option for uncomplicated malaria during the first trimester of pregnancy is being considered by WHO after the Malaria Policy Advisory Committee’s review (*2*,*3*). Women seeking care in the United States will now have a third treatment option for uncomplicated malaria during the second and third trimesters of pregnancy, and during the first trimester of pregnancy when other treatment options are unavailable, that is safe and effective for treating *P. falciparum* infections acquired in regions with chloroquine resistance. To assess the implementation and impact of these updated recommendations in the United States, data from the National Malaria Surveillance System will be used to examine how antimalarials are used to treat uncomplicated malaria in pregnant women, as well as population-specific disease burden; in addition, the FDA Adverse Event Reporting System maintains adverse event and medication error data, which can be used to monitor adverse events associated with AL use during pregnancy.
